# Group Method of Data Handling Using Christiano–Fitzgerald Random Walk Filter for Insulator Fault Prediction

**DOI:** 10.3390/s23136118

**Published:** 2023-07-03

**Authors:** Stefano Frizzo Stefenon, Laio Oriel Seman, Nemesio Fava Sopelsa Neto, Luiz Henrique Meyer, Viviana Cocco Mariani, Leandro dos Santos Coelho

**Affiliations:** 1 Digital Industry Center, Fondazione Bruno Kessler, 38123 Trento, Italy; 2 Department of Mathematics, Computer Science and Physics, University of Udine, 33100 Udine, Italy; 3 Graduate Program in Applied Computer Science, University of Vale do Itajai, Itajai 88302-901, SC, Brazil; 4 Electrical Engineering Graduate Program, Regional University of Blumenau, Blumenau 89030-000, SC, Brazil; 5 Mechanical Engineering Graduate Program, Pontifical Catholic University of Parana, Curitiba 80215-901, PR, Brazil; 6 Department of Electrical Engineering, Federal University of Parana, Curitiba 81530-000, PR, Brazil; 7 Industrial and Systems Engineering Graduate Program, Pontifical Catholic University of Parana, Curitiba 80215-901, PR, Brazil

**Keywords:** Christiano–Fitzgerald random walk filter, electrical power grids, group method of data handling, leakage current, time series forecasting

## Abstract

Disruptive failures threaten the reliability of electric supply in power branches, often indicated by the rise of leakage current in distribution insulators. This paper presents a novel, hybrid method for fault prediction based on the time series of the leakage current of contaminated insulators. In a controlled high-voltage laboratory simulation, 15 kV-class insulators from an electrical power distribution network were exposed to increasing contamination in a salt chamber. The leakage current was recorded over 28 h of effective exposure, culminating in a flashover in all considered insulators. This flashover event served as the prediction mark that this paper proposes to evaluate. The proposed method applies the Christiano–Fitzgerald random walk (CFRW) filter for trend decomposition and the group data-handling (GMDH) method for time series prediction. The CFRW filter, with its versatility, proved to be more effective than the seasonal decomposition using moving averages in reducing non-linearities. The CFRW-GMDH method, with a root-mean-squared error of $3.44\times 10^{-12}$, outperformed both the standard GMDH and long short-term memory models in fault prediction. This superior performance suggested that the CFRW-GMDH method is a promising tool for predicting faults in power grid insulators based on leakage current data. This approach can provide power utilities with a reliable tool for monitoring insulator health and predicting failures, thereby enhancing the reliability of the power supply.

## 1. Introduction

Power grid insulators are responsible for the mechanical support and electrical insulation of the conductors in low-/medium-/high-voltage overhead networks [1]. Since they are exposed to the environment and eventually adverse weather conditions, insulators must withstand the mechanical and electrical stresses and the environmental ones [2]. The external factors present in each application need to be considered when choosing the type of insulation to be applied depending on the environment, as these interactions can compromise the network’s proper performance and the insulator’s life [3].

The contamination of the insulator surface, associated with bad environmental conditions, can lead to a more-conductive surface, beginning by increasing the chance of partial discharges and rising leakage currents occurring, which can result in flashover [4]. Contamination is a present problem in places close to industries, agricultural farming, mining, seaside areas, or unpaved roads, where the incidence of rainfall is not enough to clean the surface of the insulator [5]. Therefore, in more critical locations, such as coastal regions, preventive cleaning of the insulators is necessary to reduce insulation faults in the electrical power systems [6].

For this matter, the significance of this study lied in its focus on leakage current, a key indicator of insulator contamination [7]. By monitoring this effect, we can predict failures in the electrical power network [8]. This paper proposes a novel approach to this issue: a hybrid model that combines the group method of data handling (GMDH) with the Christiano–Fitzgerald random walk (CFRW) filter for predicting the increase in leakage current. This method was compared to the long short-term memory (LSTM) model, and the CFRW was evaluated against the seasonal decomposition using moving averages (SDMA).

The main contributions of this research are:

- The application of the Christiano–Fitzgerald random walk filter for noise mitigation in the context of power grid insulator contamination.
- The group method of data handling has shown less time needed for training and superior performance to the LSTM.
- The development of a hybrid method for time-series-based failure prediction, focusing on evaluating the increasing trend of leakage current.

The remainder of this paper is structured as follows: Section 2 presents related works regarding time series forecasting. Section 3 covers the description of the problem and the performed laboratory analysis. In Section 4, the proposed method is presented, and the results are evaluated in Section 5. Section 6 draws a conclusion and discusses future research directions.

## 2. Related Works

Given the need to keep the electric power system running, techniques for the maintenance and prediction of insulator failure are employed by electric power utilities [9]. One of the most-common techniques is visual inspection, which can be further improved using thermographic cameras [10], ultraviolet light detectors [11], ultrasound signals [12], radio interference, acoustic techniques [13], unmanned aerial vehicles [14], and leakage current techniques [15]. The maintenance is carried out by field technicians, which, when detecting possible defective insulators, perform the cleaning or the replacement of the insulator [16].

According to Yang et al. [17], image processing, especially based on deep architecture, is becoming popular. Additionally, image preprocessing is a way to improve the classifier models [18]. The use of artificial-intelligence (AI)-based methods is a promising alternative for power system monitoring and can even be applied to assess the level of contamination of the power grids.

Salem et al. [19] presented a work where the flashover voltage of a porcelain insulator was investigated concerning the density of the salt deposit. Besides AI applications, the performance of insulators has been explored by using advanced methods such as the finite element method, as presented by Ahmed et al. [20] for the evaluation of polluted environments for polymeric insulators and by Stefenon et al. [21] for the design of insulators.

In the paper of Salem et al. [22], the support vector machine was employed to forecast the deterioration of the room-temperature vulcanized coatings on contaminated glass insulators. Time series forecasting has been applied in several fields, for issues related to financial [23], security [24], energy price [25], traffic flow [26], and epidemiology [27], among others. Considering that leakage current is a strong indication that flashovers may occur, evaluating its evolution concerning time series analysis is a promising alternative and will be the focus of this paper.

Choosing the appropriate model to perform the prediction is a difficult task, where deep-learning-based models may have a superior ability to handle non-linear data and shallow models typically have lower computational effort and can have acceptable prediction results [28]. Combined structures such as the adaptive neuro-fuzzy inference system (ANFIS) have the smoothness of fuzzy systems and the adaptive characteristic of neural nets [29]; therefore, these are also an alternative in this context.

For time series forecasting, LSTM has been increasingly applied. LSTMs overcome the vanishing gradient problem by incorporating a memory cell and several gating mechanisms. The memory cell allows the network to retain information over long sequences, and the gating mechanisms control the information flow in and out of the cell [30]. Due to this characteristic, this model is promising for predicting failures, being applied in benchmarks along with ensemble learning models, ANFIS, and the GMDH [31].

According to Branco et al. [32], using filters for denoising is necessary when the considered signal has high non-linearities. In their work, the wavelet transform was combined with LSTM for fault forecasting considering the number of alarms of the distribution power branches of an electric utility company. The results showed that, without the wavelet, the model could not predict the variation of the faults over time with an acceptable error.

The ensemble learning models have been explored due to their high efficiency; several architectures based on this approach have been used for time series forecasting, such as the cooperative [33], stacking [34], heterogeneous [35], bagging [36], boosting [37], random subspace [38], and random forest [39] ensemble learning models. The advantage of this approach is the combination of simpler models to build a stronger model [40], which has a high predictive ability and can be more efficient than models based on deep learning.

The GMDH is a promising approach for time series fault forecasting; due to its adaptive features, it can use an optimized structure defining the neurons during the training, excluding the neurons when the worst predictions are achieved. Combining the GMDH with noise-reducing methods such as the wavelet transform may improve the network, outperforming well-established models such as LSTM and ANFIS [41]. Due to the advantages of applying filters for noise reduction in time series, several authors have explored hybrid methods that combine filters with prediction models.

A Hodrick–Prescott (HP) filter-based modeling, which identified repeated high and low structural characteristics around a given carbon price, was proposed by Qin et al. [42], overcoming the parallel series hybridization obstacle with respect to identifying linear and non-linear models. The work presented by Klarl [43] using a continuous regression method found that the elasticity of emissions to the gross domestic product (GDP) was not constant over time, regardless of the filtering technique employed, such as the HP, the Christiano–Fitzgerald, the Baxter–King, or the Butterworth filters. Environmental policy instruments that do not prove to be suboptimal must consider this asymmetric emissions response due to variations in the GDP.

For the long-term seasonal component (LTSC), the models based on wavelet are suitable to extract the LTSC of a series of values and are more accurate for predicting values up to one year ahead, but are highly complex models. To improve the forecasting technique, the HP filter was proposed in the paper of Weron and Zator [44], to identify the LTSC in the price of electricity. Extended multi-reservoir echo state network models were proposed with the HP filter for time series forecasting by Li, Liu, and Tanaka [45]. The HP filter was applied recursively to decompose the time series data into several trend and cycle components.

Dutra, Dias, and Teixeira [46] identified the most-suitable way to detect financial cycles, such as the gross domestic product (GDP) by analyzing four financial variables: credit, real estate prices, stock prices, and interest rates. The Christiano–Fitzgerald filter is applied to estimate the cycles from the time series. Some recommendation systems have attempted to capture the complexity of interactions between user and item resources to obtain reliable recommendations. Lee and Kim [47] proposed a recommendation system using the external feature product matrix and cross-convolutional filters, alleviating the overfitting problem.

Apaydin et al. [48] investigated the application of seasonal trend decomposition based on loess (STL) and attribute selection preprocessing methods in forecasting monthly river flows. The hybrid models recorded higher accuracy than other independent models even without preprocessing. Tebong et al. [49] used deep learning models to create ensembles. STL decomposition decomposed reservoir inflows and precipitation into random, seasonal, and trend components. The ensemble models were evaluated using decomposed data of daily inflows and precipitation from a reservoir, with the multivariate STL-dense model being the best.

In the study of Qin, Li, and Li [50], two hybrid approaches that combine STL with the echo state network enhanced by the grasshopper optimization method and adaptive reinforcement model were proposed to predict the flow of passengers in China per month. The results showed that, by using STL, higher accuracy was obtained compared to other prediction methods. According to the authors mentioned so far, filters for pre-processing are a promising approach in time series analysis, and it was explored in this paper.

Everything considered, in the context of power grid insulators, leakage current, a strong indicator of potential flashovers, is a promising focus for time series analysis. However, choosing the appropriate prediction model is challenging, with deep-learning-based and shallow models each having advantages [28].

This paper proposes a novel approach to this issue, combining the GMDH with the CFRW filter for predicting the increase in leakage current. This hybrid method is a promising alternative for time-series-based failure prediction, focusing on evaluating the increasing trend of leakage current, which is the issue to be analyzed.

## 3. Problem Description and Laboratory Analysis

When dry, the contamination layer deposited on the insulators is usually not highly conductive, although with moisture (rain, fog), its conductivity might increase [51]. Increasing the conductivity, partial discharges occur more often and have greater intensity, evolving to the formation of a leakage current [52], which could evolve until a complete breakdown, known as flashover [53]. Failure reduces reliability in the power grid, and it is challenging to identify insulators that have lost their insulating properties [54].

This contamination process and increased leakage current can take years to cause insulator failure [55]. This work accelerated this process to evaluate the insulator’s endurance to contamination. This section presents how the laboratory experiments, which originated the database that was used for training and testing the proposed model, were obtained, to allow the reproduction of the experiment for future comparisons.

**Figure 1:**
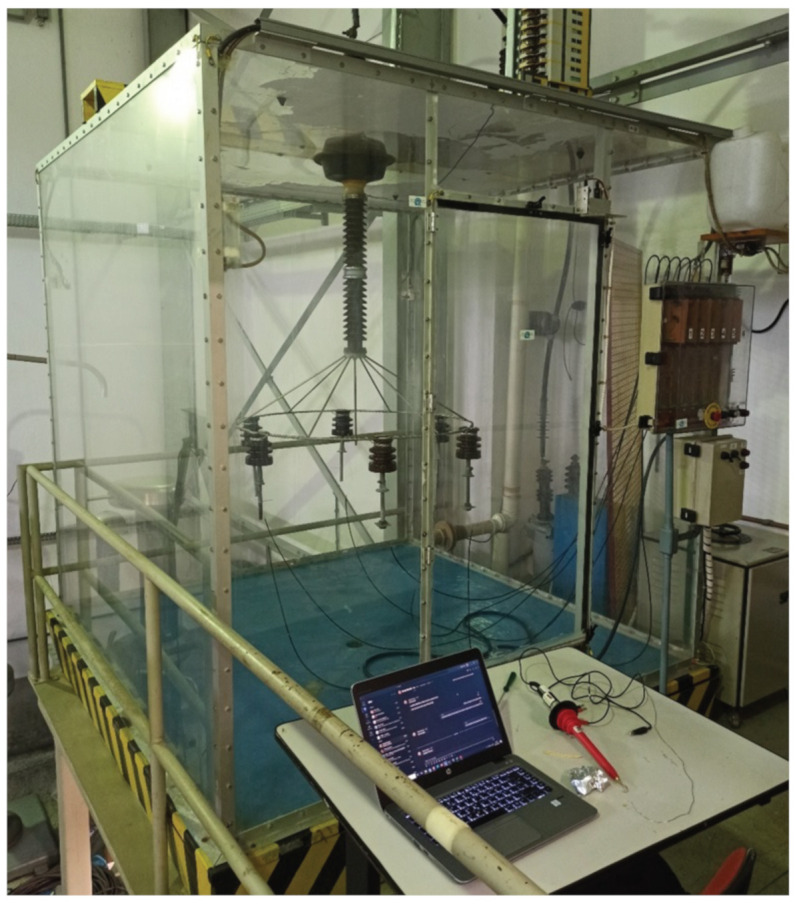
Salt fog chamber (high-voltage laboratory).

To compose the database that were used in the present paper, tests were performed in the salt fog chamber (see Figure 1), in the high-voltage laboratory at the Regional University of Blumenau, Brazil. The salt fog chamber test consists of simulating the behavior of commercial insulators under controlled conditions. The insulators were exposed to the rated design voltage and salt fog in this test. The amount of salt in the water that was sprayed to generate the fog was controlled. During the test, there was a gradual increase in the amount of salt deposited on the surface of the insulator, until the dielectric breakdown occurred.

The salt fog chamber had dimensions of 2 m × 2 m × 2 m, with a variable voltage transformer and a power transformer of 15 kVA to perform the experiments. The chamber had four foggers, one in each upper corner, and a 1m-diameter metal ring connected to the transformer through a bushing, to apply voltage to the insulators to be tested. The insulators were connected to the central ring through a mooring system like those used for insulators in distribution lines. The step-up transformer, controlled by a variable-voltage transformer, raised the voltage, which was connected to the bushing in the chamber.

The complete experiment was conducted over 6 days, with intermittent exposure, totaling 28 h of effective exposure. The test started with a low concentration of salt, then gradually increased until it reached a very high concentration to contaminate the insulators to the point of causing a flashover. When the flashover occurred, the current tended to reach extremely high values, limited to 200 mA by a fuse, and the measurement was ended at the insulator to which the discharge occurred.

**Figure 2:**
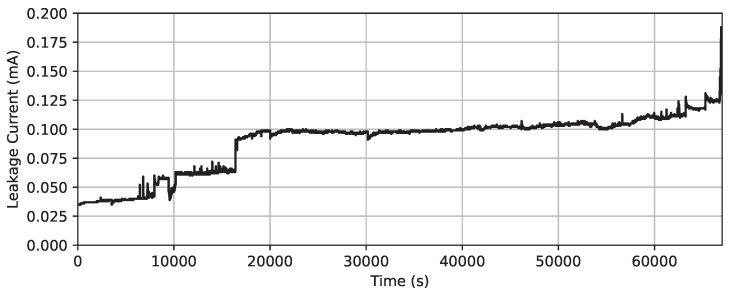
Original signal: Measurement of the leakage current over time. At the end, the insulator had more than 200 mA and the flashover occurred.

In this research, among six insulators of 15 kV, two had no dielectric breakdown; these could not be used, because the disruptive failure did not occur. Considering the samples with a flashover, the insulator that had the longest dielectric breakdown was used. From the total time of the effective exposure, the considered sample had the flashover after 18.62 h (67,040 s), and the variation of the leakage current measurement from this insulator is presented in Figure 2. Contamination accumulated on the surface of the insulator in a random and distributed manner, as occurs in the field; for this reason, there was a difference between the dielectric breakdown point of different insulators.

## 4. Methodology

This paper proposes a hybrid method based on combining the GMDH with the CFRW filter. The time series prediction was performed through the GMDH, and the CFRW filter was applied to reduce noise and unrepresentative variations. The proposed method, named CFRW-GMDH, will be explained in this section, as well as the methods that were compared to validate the proposed model.

### 4.1. Group Method of Data Handling

**Figure 3:**
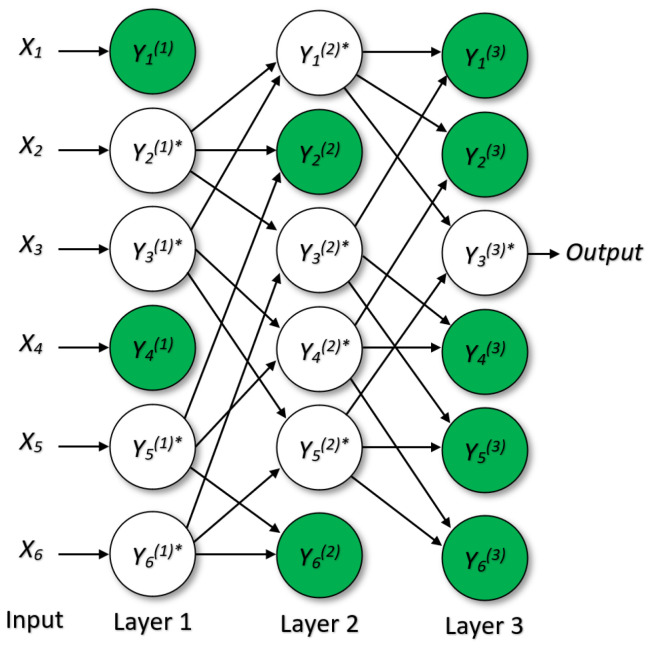
Illustration of the GMDH architecture.

The GMDH is an inductive self-organizing iterative algorithm that utilizes polynomial models [56]. Its fundamental principle is to generate many models, assess each according to a specific criterion, and select the optimal model [57]. This operation involves incrementally adding layers of nodes, where each node represents a two-input function that is fit using a polynomial of a given degree, as depicted in Figure 3.

Only a layer’s top-performing results (depicted as white neurons in Figure 3) are passed on to the next. Neurons that yield less-accurate predictions (illustrated as green neurons in Figure 3) are eliminated in the process, thereby optimizing the structure. The optimization of the structure requires the specification of the maximum number of neurons and layers; these network hyperparameters will be evaluated in this paper.

Given a time series, the GMDH learns the relationships between the time lags and then automatically determines the optimal path. The GMDH’s mapping of the input and output variables constitutes a non-linear function, given by:

(1) $$\hat{y}\left(x_{1},\ldots ,\;x_{n}\right)=a_{0}+\sum_{i=1}^{n}a_{i}x_{i}+\sum_{i=1}^{n}\sum_{j=1}^{n}a_{ij}x_{i}x_{j}+\ldots +\sum_{i=1}^{n}\sum_{j=1}^{n}\sum_{k=1}^{n}a_{ijk}x_{i}x_{j}x_{k}$$

where $x_{i}$ and $x_{j}$ denote the input variables and *n* is the number of considered samples.

The coefficients are estimated using a regression approach for the pair of input variables ($x_{i},x_{j}$) as follows:

(2) $$G\left(x_{i},\;x_{j}\right)=a_{0}+a_{1}x_{i}+a_{2}x_{j}+a_{3}x_{i}^{2}+a_{4}x_{j}^{2}+ax_{i}x_{j}.$$

in which *y* signifies the observed value, $\hat{y}$ the predicted value, and *w* the result of the external criterion, which is given by:

(3) $$w=\frac{\sum_{n=1}^{P}{\left({\hat{y}}_{n}-y_{n}\right)}^{2}}{\sum_{n=1}^{P}{\left(y_{n}\right)}^{2}}.$$

where *P* denotes the number of test sets. If *w* does not decrease with an earlier layer, it suggests that the model’s prediction error is not declining, thus terminating the model expansion and generating the results [58].

The coefficients in the polynomial function were computed via the least-squares error (LSE) method. This mathematical technique aims to minimize the sum of the squares of the residuals, thus reducing the difference between *y* and $\hat{y}$. The procedure of this fitting method is described as follows:

(4) $$LSE=\left\{\begin{array}{c} \hat{y}\left(x_{1},\ldots ,\;x_{n}\right)=G\left(x_{i},\;x_{j}\right)\\ e=\sum_{n=1}^{N}{\left(y-\hat{y}\right)}^{2}\\ \frac{de}{da_{k}}=0,\;\;k=1,\;2,\;3,\;4,\;5. \end{array}\right.$$

To streamline the analysis, the results are presented in matrix form:

(5) $$a={\left(X^{T}X\right)}^{-1}X^{T}y$$

where,

(6) $$X=\left\{\begin{array}{cccccc} 1 & x_{i1} & x_{j1} & x_{i1}x_{j1} & x_{i1}^{2} & x_{j1}^{2}\\ 1 & x_{i2} & x_{j2} & x_{i2}x_{j2} & x_{i2}^{2} & x_{j2}^{2}\\ 1 & x_{i3} & x_{j3} & x_{i1}x_{j2} & x_{i3}^{2} & x_{j3}^{2}\\ \vdots & \vdots & \vdots & \vdots & \vdots & \vdots \\ 1 & x_{in} & x_{jn} & x_{in}x_{jn} & x_{in}^{2} & x_{jn}^{2} \end{array}\right\}.$$

Following this, the Christiano–Fitzgerald random walk and the seasonal decomposition using moving averages filters are explained.

### 4.2. Christiano–Fitzgerald Random Walk Filter

The CFRW filter is an econometric technique that offers a more-adaptable strategy for analyzing time series data, particularly in cases where variables exhibit stochastic trends or nonstationary behavior [59]. The method aims to approximate the trend constituent of a time series, especially when there is a need for more information regarding the actual characteristics of the underlying process [60].

The CFRW filter is applicable to any univariate time series, $y_{t}$, that can be represented as a random walk. The random walk model assumes that the change in $y_{t}$ from one period to the next, $y_{t}-y_{t-1}$, is a random variable with a mean of zero [61]. A random walk process can be represented as:

(7) $$\begin{array}{c} y_{t}=y_{t-1}+\varepsilon_{t} \end{array}$$

where $\varepsilon_{t}$ is a stochastic error term considered independent and identically distributed with zero mean and constant variance. Given a sample $y_{t}$${}_{t=1}^{T}$, the goal is to obtain an estimate (${\hat{y}}_{t}$) of the unobserved component of the time series (trend) [56].

This procedure involves projecting $y_{t}$ onto the space of *d*-step-ahead and *d*-step-behind linear predictions, where *d* is a bandwidth parameter that should be selected in advance [62]. The CFRW filter is a linear projection:

(8) $$\begin{array}{c} {\hat{y}}_{t}=\sum_{j=-d}^{d}\omega_{j}y_{t+j} \end{array}$$

in which $\omega_{j}$ are weights determined by the minimization problem and depend on *d* and the autocorrelation structure of $y_{t}$. If $y_{t}$ is a random walk, then the weights $\omega_{j}$ converge to those of an ideal low-pass filter as d→∞ [43].

### 4.3. CFRW-GMDH Hybrid Method

Algorithm 1 presents the steps involved in the proposed CFRW-GMDH method, a hybrid approach combining the CFRW filter and the GMDH.
**Algorithm 1:** CFRW-GMDH Hybrid Method
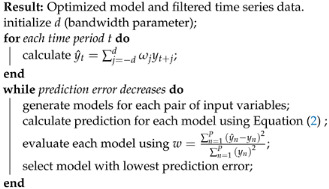


For comparative purposes, the SDMA filter was compared in this paper to the CFRW filter, and the SDMA method is explained in the following subsection.

### 4.4. Seasonal Decomposition using Moving Averages

The SDMA is a statistical technique for decomposing time series data into their trend, seasonal, and residual components, as well as seasonal trend decomposition based on locally estimated scatterplot smoothing (STL) [63], which aims to identify patterns and seasonality in the data and separate them from underlying trends or aleatory variations [64]. The trend component ($t_{t}$) is given by applying a weighted moving average to the original signal, according to:

(9) $$\begin{array}{c} t_{t}=\frac{\sum_{i=1}^{m}w_{i}y_{t-m+i}}{\sum_{i=1}^{m}w_{i}} \end{array}$$

where $w_{1},w_{2},\ldots ,w_{m}$ are the weights defining the smoothing function and *m* is the length of the moving average window. The residual component ($r_{t}$) is achieved by subtracting the trend from the original data, given by:

(10) $$\begin{array}{c} r_{t}=y_{t}-t_{t}. \end{array}$$

The filter eliminates the high frequency, and the smoothed signal is subtracted from the original to have the residual component, corresponding to any high-frequency fluctuations left out of the moving average [65]. The seasonal component ($s_{t}$) is calculated by averaging the residuals across a defined window, corresponding to the length of the seasonal cycle, as the following:

(11) $$\begin{array}{c} s_{t}=\frac{\sum_{i=t-P+1}^{t}r_{i}}{P} \end{array}$$

where *P* is the length of the seasonal cycle. Then, the decomposition is reconstructed by adding its components accordingly:

(12) $$\begin{array}{c} y_{t}=t_{t}+s_{t}+r_{t}. \end{array}$$

In this paper, regarding the use of the filter, the prediction of the signal was performed with respect to the trend. Therefore, $s_{t}$ and $r_{t}$ were not considered. Here, LSTM was used for the benchmarking, and a brief explanation of this model is given in the subsequent subsection.

### 4.5. Long Short-Term Memory

LSTM is a recurrent neural network (RNN) that captures long-term dependencies in sequential data [66]. One of the main advantages of LSTM over traditional RNNs is its ability to maintain and control the information flow through a memory cell [67]. The memory cell allows LSTM to selectively remember or forget information in long sequences, which helps overcome the vanishing gradient problem commonly encountered in training RNNs [68].

LSTMs achieve this memory control through the use of specialized units called gates. These gates, which include the input gate ($i_{t}$), forget gate ($f_{t}$), and output gate ($o_{t}$), regulate the flow of information into, out of, and within the memory cell [69]. $f_{t}$ determines what information should be discarded from the cell; $i_{t}$ controls the addition of new information to the cell; $o_{t}$ decides which information should be exposed to the next layer of the network [70]. LSTM can be given by:

(13) $$\begin{array}{c} i_{t}=\sigma_{g}\left(W_{i}x_{t}+R_{i}h_{t-1}+b_{i}\right),\\ f_{t}=\sigma_{g}\left(W_{f}x_{t}+R_{f}h_{t-1}+b_{f}\right),\\ o_{t}=\sigma_{g}\left(W_{o}x_{t}+R_{o}h_{t-1}+b_{o}\right). \end{array}$$

where *R* and *W* are earnings matrices and *b* is the polarization matrix.

The architecture of an LSTM cell consists of these gates, a memory cell, and various activation functions ($\sigma_{g}$) [71]. The cell operates sequentially, taking an input at each time step, updating its memory content, and generating an output [72]. This makes LSTMs well-suited for processing and modeling sequential data such as time series [73], which was the focus of this paper.

### 4.6. Experiment Setup

The experiments were implemented in MATLAB and computed using an i5-7300HQ with 20 GB of random access memory and a graphics processing unit NVIDIA GeForce GTX 1050 Ti. The root-mean-squared error (RMSE), mean-squared error (MSE), mean absolute percentage error (MAPE), mean absolute error (MAE), and coefficient of determination (R${}^{2}$) were evaluated, given by:

(14) $$\mathrm{RMSE}=\sqrt{\frac{1}{n}\sum_{i=1}^{n}{\left(y_{i}-{\hat{y}}_{i}\right)}^{2}}$$

(15) $$\mathrm{MSE}=\frac{1}{n}\sum_{i=1}^{n}{\left(y_{i}-{\hat{y}}_{i}\right)}^{2}$$

(16) $$\mathrm{error}=\frac{1}{n}\sum_{i=1}^{n}\left|\frac{y_{i}-{\hat{y}}_{i}}{y_{i}}\right|\times 100$$

(17) $$\mathrm{MAE}=\frac{1}{n}\sum_{i=1}^{n}\left(y_{i}-{\hat{y}}_{i}\right)$$

(18) $$R^{2}=1-\frac{\sum_{i=1}^{n}{\left(y_{i}-{\hat{y}}_{i}\right)}^{2}}{\sum_{i=1}^{n}{\left(y_{i}-{\bar{y}}_{i}\right)}^{2}}$$

where *n*, *y*, and $\hat{y}$ were previously defined. The $\bar{y}$ is the average of the observed value.

## 5. Experiments and Discussion

The variation in the training data percentage can influence the model’s performance. Therefore, an initial evaluation is presented in Table 1 to assess the impact of this variation. Initially, a maximum of 50 neurons in up to three layers were considered, and then, each hyperparameter was evaluated. In this section, the best results are highlighted in bold.

Using a lower data value for training generally resulted in lower performance results regarding the error, making the model faster to be trained. The best ratio between the data to train and test the model was using 70% of data for training and 30% of data for testing the model. Therefore, this ratio was considered for all analyses presented in this paper. In Table 2, the impact of using a higher maximum value of layers on the configuration of the structure is evaluated.

Increasing the number of layers led to a shorter time required to compute the model; however, it did not reflect progressive improvements in the results, considering the input data used here. The best results regarding lower error were obtained using two (RMSE and MSE) and three layers (MAPE and MAE). The processing time was not a value to be optimized in this evaluation. Considering that using two layers may result in limited flexibility for the model to adapt in the GMDH, three layers were used as the standard architecture. A detailed analysis of the definition of the maximum number of neurons is presented in Table 3.

Using three layers, the network became stable with a maximum of 50 neurons, and thus, the processing time was similar even when using a considerably higher maximum number of neurons. When more layers were used, the model tended to increase the computational effort considerably in preliminary evaluations, reflecting the time required to compute the analysis. Despite a small improvement in the RMSE and MSE using more neurons, the model stabilized between 45 and 50 neurons during the simulation, sufficient to achieve acceptable error results.

**Figure 4:**
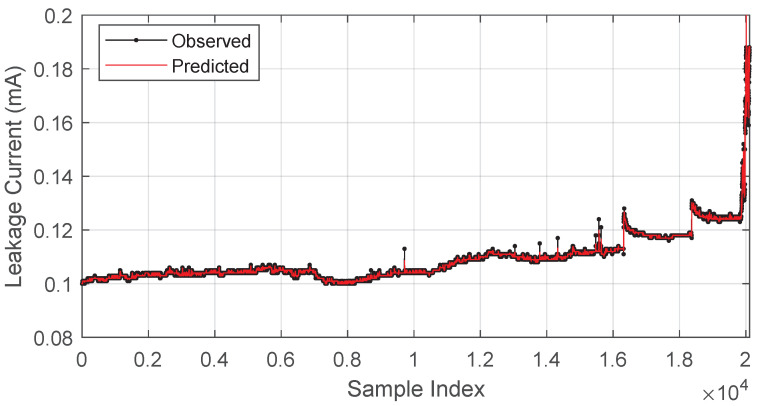
Original versus predicted signals (non-filtered).

Based on the initial evaluation of the maximum values of the hyperparameters, the GMDH proved to be efficient, since it reached acceptable prediction values, converging in a short period. The result of this prediction concerning the original signal is presented in Figure 4. The next section presents the evaluation and discussion of the filter application.

### 5.1. Filter Evaluation

Reducing unrepresentative high-frequency noise is the first step in the time series analysis evaluated in this paper. Considering the use of the CFRW filter, three hyperparameters can be adjusted to adapt this filter to the filtering needs of the signal. These hyperparameters were the minimum period of oscillations, the maximum period of oscillations, and the drift (whether or not to remove a trend from the data).

The removal of the trend resulted in a signal that lost the characteristics that were considered in this paper since the trend was the main indicator of increased leakage current; for this reason, this hyperparameter was considered equal to false. When it was not necessary to evaluate the trend of the signal, only its variation, the drift hyperparameter, may be considered, being applied to evaluate abrupt variations of the signal.

The increase in the oscillation period’s minimum value did not improve the filtered signal; therefore, this hyperparameter was considered equal to two because this was the minimum value for the filter to be applied. Signals with other properties may have a greater influence on the variation of the minimum period of oscillations. In this case, the increase in this hyperparameter resulted in a greater filter signal disparity than the original signal.

**Figure 5:**
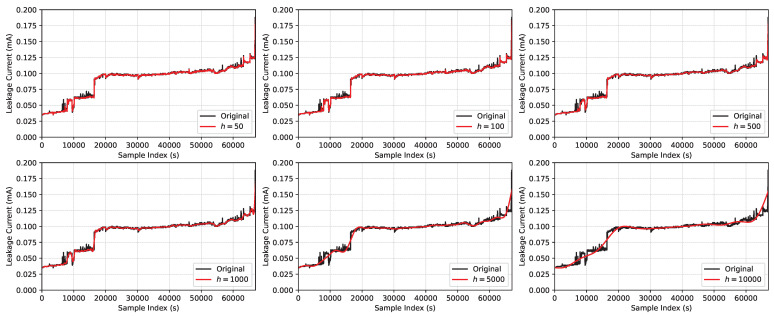
Trend of the Christiano–Fitzgerald random walk filter vs. original signal.

The hyperparameter that had a major influence on the filtering was the definition of a maximum period of oscillations (*h*), where values under 50 were not enough to filter the signal and values higher than 1000 resulted in a filtered signal that lost its properties of variation; the influence of this hyperparameter value is presented in Figure 5.

The SDMA filter is an alternative to decomposing the signal and has its trend with less noise. The problem with this filter is that there is no flexibility in adjusting the method to suit the needs of the signal. If the signal has a high incidence of high-frequency noise with relevant information, this filter became even less suitable based on the experiments’ results. The possibilities of varying the SDMA configuration can be realized using the type of seasonal component, which can be either “additive” or “multiplicative”. In this work, both had equivalent results. Moreover, the other variation possibilities did not result in more flexibility in this architecture; therefore, the CFRW filter was more suitable in this initial analysis.

**Figure 6:**
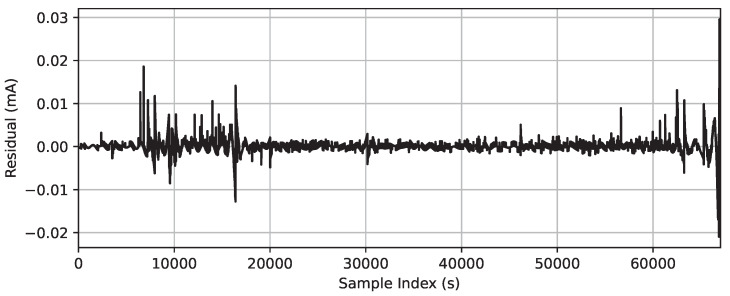
Residual of the signal of the CFRW filter (h=1000).

A value of *h* equal to 1000 was set for comparative purposes since, by using larger values, significant variants were lost, which may be relevant in this analysis. Based on this configuration, the GMDH was evaluated from the signal filtering. Besides the trend of the signal used for the prediction, the CFRW filter gave the residual of the signal presented in Figure 6, indicating where the leakage current had more variation. In this case, the major variation of the residual of the signal happened just before the flashover.

Table 4 presents the statistical evaluation of the use of the CFRW filter in relation to the original GMDH. Using the CFRW, the GMDH became considerably higher, with a low error in all metrics evaluated compared to the original GMDH; this evaluation was performed by initializing the network with random persons in 50 runs, to validate the robustness of the proposed method with respect to the variability of several simulations.

Since promising results were obtained using the CFRW filter combined with the GMDH, and benchmarking was performed to compare the proposed method (CFRW-GMDH) with the original LSTM and against LSTM using the CFRW filter.

### 5.2. Benchmarking Evaluation

The comparative analysis presented in Table 5 showed that the proposed method, besides being superior in having a lower prediction error, had lower computational effort than the LSTM. Using the CFRW filter improved the performance of LSTM, proving that the hybrid approach excelled over the standard models. However, both LSTM and the CFRW-LSTM were inferior to the proposed model.

**Figure 7:**
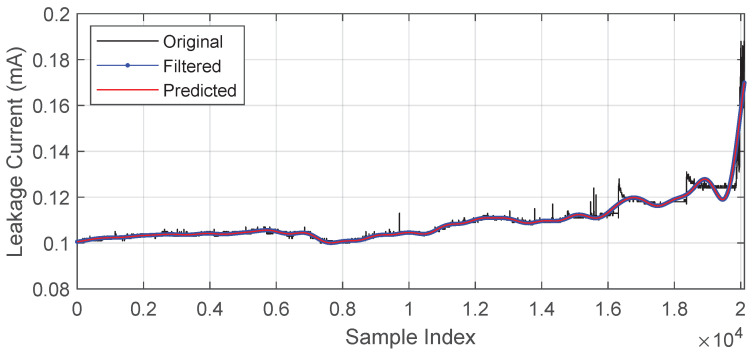
Original, filtered (observed), and predicted signals.

The original signal, the filtered signal, and the one-step-ahead prediction of the filtered signal are shown in Figure 7. The proposed method proved effective enough to have a visual overlap between the predicted and observed signals, which in this case was the signal after using the CFRW filter.

## 6. Conclusions

Since they are essential parts of electrical systems, insulators are exposed to various external environmental factors that could reduce their effectiveness. The selection of insulators and their maintenance are crucial elements to consider due to the complexity of these factors. Cleaning in key spots has effectively prevented difficulties such as pollution, which can degrade the conductivity of the surface of the insulators and can cause electrical discharges and flashovers.

This study presented a hybrid method that combines the Christiano–Fitzgerald random walk filter and the GMDH to forecast the rise in leakage current, a crucial sign of insulator contamination. The filter is instrumental in mitigating noise and adapting to the specific requirements of an application. Additionally, it was shown that the GMDH outperformed the LSTM model in terms of efficiency, requiring less training time.

Experimental investigation into the GMDH model presented several key insights. The model’s performance appeared to be directly influenced by the percentage of data used for training. Lower data percentages for training generally resulted in decreased error performance, but made the model faster to train. The most-optimal balance between the data used for training and testing was found at a 70–30% ratio. This configuration was adopted for the entirety of the analyses.

Analyzing the impact of varying the maximum number of layers, it was observed that increasing the number of layers shortened the computation times, but did not necessarily translate into progressive performance improvements. The most-minimal errors were achieved with two (RMSE and MSE) and three layers (MAPE and MAE). To preserve model adaptability, a three-layer architecture was decided as the standard.

When exploring the optimal number of neurons, the network was found to stabilize with a maximum of 50 neurons. The processing time remained consistent even when a higher maximum number of neurons was applied. Despite minor improvements in the RMSE and MSE using more neurons, the model reached a stabilization point between 45 and 50 neurons, ensuring acceptable error results.

The experiments also highlighted the efficiency of the GMDH model. It converged quickly while achieving satisfactory prediction values. In terms of the filter application, it was possible to conclude that the Christiano–Fitzgerald random walk (CFRW) filter was the most-effective in reducing unrepresentative high-frequency noise. Its hyperparameters allowed for adaptations according to the filtering needs of the signal. The CFRW filter also helped improve the GMDH model substantially, resulting in significantly lower errors in all metrics evaluated compared to the original GMDH.

The proposed hybrid approach is a viable alternative for forecasting time-series-based failure. It pays close attention to the growing leakage current trend, a crucial area that needs to be examined. Therefore, this method can be applied to manage power systems in a predictive maintenance and effective decision-making manner. To apply the proposed method in the field, it needs a specialized team to employ it considering that the measurement is connected to the grid.

The potential of the suggested method can be further investigated in various environmental scenarios and settings in the future, improving its adaptability and generalizability. In addition, comparable approaches can be used for various predictive indicators within the power system, even though this work concentrated on leakage current prediction. Another exciting area for future research is the scalability of the suggested technique in terms of larger datasets and various insulator kinds.

## Figures and Tables

**Table 1:** Results of the variation in the percentage of data for training and testing the model.

Train_Test (%)	RMSE	MSE	MAPE${}_{\%}$	MAE	R${}^{2}$	Time (s)
50_50	6.33 $\times \;10^{-3}$	4.01 $\times \;10^{-5}$	5.94 $\times \;10^{-2}$	9.24 $\times \;10^{-5}$	0.4174	**5.53**
60_40	1.10 $\times \;10^{-3}$	1.22 $\times \;10^{-6}$	7.17 $\times \;10^{-3}$	1.45 $\times \;10^{-5}$	0.9828	7.56
70_30	**6.65 $\times \;10^{-4}$**	**4.42 $\times \;10^{-7}$**	**4.63 $\times \;10^{-3}$**	**8.29 $\times \;10^{-6}$**	**0.9945**	9.03
80_20	7.91 $\times \;10^{-4}$	6.26 $\times \;10^{-7}$	1.40 $\times \;10^{-2}$	2.05 $\times \;10^{-5}$	0.9936	10.10
90_10	1.01 $\times \;10^{-3}$	1.02 $\times \;10^{-6}$	2.85 $\times \;10^{-2}$	4.47 $\times \;10^{-5}$	0.9901	12.75

**Table 2:** Results of the variation in the number of layers.

Layers	RMSE	MSE	MAPE${}_{\%}$	MAE	R${}^{2}$	Time (s)
2	**6.21 $\times \;10^{-4}$**	**3.86 $\times \;10^{-7}$**	1.82 $\times \;10^{-2}$	2.75 $\times \;10^{-5}$	**0.9952**	**0.89**
3	6.50 $\times \;10^{-4}$	4.22 $\times \;10^{-7}$	**9.43 $\times \;10^{-3}$**	**1.43 $\times \;10^{-5}$**	0.9947	9.30
4	9.96 $\times \;10^{-2}$	9.92 $\times \;10^{-3}$	4.48 $\times \;10^{-1}$	7.46 $\times \;10^{-4}$	-	18.18
5	1.67 $\times \;10^{-3}$	2.80 $\times \;10^{-6}$	2.33 $\times \;10^{-2}$	4.10 $\times \;10^{-5}$	0.9651	27.64
6	1.90 $\times \;10^{-2}$	3.60 $\times \;10^{-4}$	4.46 $\times \;10^{-1}$	7.71 $\times \;10^{-4}$	-	37.80

**Table 3:** Results of the variation in the number of neurons.

Neurons	RMSE	MSE	MAPE${}_{\%}$	MAE	R${}^{2}$	Time (s)
5	7.02 $\times \;10^{-4}$	4.92 $\times \;10^{-7}$	1.89 $\times \;10^{-2}$	2.77 $\times \;10^{-5}$	0.9939	**0.77**
10	6.51 $\times \;10^{-4}$	4.23 $\times \;10^{-7}$	1.18 $\times \;10^{-2}$	1.85 $\times \;10^{-5}$	0.9947	1.22
50	6.47 $\times \;10^{-4}$	4.18 $\times \;10^{-7}$	1.08 $\times \;10^{-2}$	1.54 $\times \;10^{-5}$	0.9948	9.22
100	6.24 $\times \;10^{-4}$	3.89 $\times \;10^{-7}$	1.33 $\times \;10^{-2}$	1.91 $\times \;10^{-5}$	0.9952	10.14
500	6.19 $\times \;10^{-4}$	3.84 $\times \;10^{-7}$	1.20 $\times \;10^{-2}$	1.74 $\times \;10^{-5}$	0.9952	9.73
1000	**6.14 $\times \;10^{-4}$**	**3.76 $\times \;10^{-7}$**	**8.55 $\times \;10^{-3}$**	**1.36 $\times \;10^{-5}$**	**0.9953**	9.22
5000	6.61 $\times \;10^{-4}$	4.37 $\times \;10^{-7}$	1.36 $\times \;10^{-2}$	1.97 $\times \;10^{-5}$	0.9946	9.05

**Table 4:** Statistical evaluation of the use of the CFRW filter on the GMDH.

Method	Measure	Mean	Median	Std Deviation	Variance
Standard GMDH	RMSE	1.59 $\times \;10^{-3}$	6.94 $\times \;10^{-4}$	2.59 $\times \;10^{-3}$	6.72 $\times \;10^{-6}$
	MSE	9.11 $\times \;10^{-6}$	4.82 $\times \;10^{-7}$	3.32 $\times \;10^{-5}$	1.11 $\times \;10^{-9}$
	MAPE${}_{\%}$	2.13 $\times \;10^{-2}$	1.33 $\times \;10^{-2}$	2.58 $\times \;10^{-2}$	6.67 $\times \;10^{-4}$
	MAE	3.29 $\times \;10^{-5}$	2.00 $\times \;10^{-5}$	4.21 $\times \;10^{-5}$	1.77 $\times \;10^{-9}$
CFRW- GMDH	RMSE	**3.42 $\times \;10^{-12}$**	**3.44 $\times \;10^{-12}$**	**1.39 $\times \;10^{-13}$**	**1.93 $\times \;10^{-26}$**
	MSE	**1.17 $\times \;10^{-23}$**	**1.18 $\times \;10^{-23}$**	**9.46 $\times \;10^{-25}$**	**8.96 $\times \;10^{-49}$**
	MAPE${}_{\%}$	**7.35 $\times \;10^{-10}$**	**7.35 $\times \;10^{-10}$**	**3.18 $\times \;10^{-11}$**	**1.01 $\times \;10^{-21}$**
	MAE	**9.21 $\times \;10^{-13}$**	**9.22 $\times \;10^{-13}$**	**4.20 $\times \;10^{-14}$**	**1.76 $\times \;10^{-27}$**

**Table 5:** Benchmarking evaluation.

Model	RMSE	MSE	MAPE${}_{\%}$	MAE	R${}^{2}$	Time (s)
Standard LSTM	3.24 $\times \;10^{-3}$	1.05 $\times \;10^{-5}$	1.61	1.90 $\times \;10^{-3}$	0.8696	305.57
CFRW- LSTM	3.02 $\times \;10^{-3}$	9.15 $\times \;10^{-6}$	1.20	1.47 $\times \;10^{-3}$	0.8819	304.86
Standard GMDH	7.93 $\times \;10^{-4}$	6.29 $\times \;10^{-7}$	1.41 $\times \;10^{-2}$	1.80 $\times \;10^{-5}$	0.9922	**8.83**
Proposed method	**3.44 $\times \;10^{-12}$**	**1.18 $\times \;10^{-23}$**	**7.42 $\times \;10^{-10}$**	**9.31 $\times \;10^{-13}$**	**1.0000**	9.17

## Data Availability

For future comparisons, the dataset is available at: https://github.com/SFStefenon/LeakageCurrent (accessed on 10 June 2023).
